# Effect of surface modification and laser repetition rate on growth, structural, electronic and optical properties of GaN nanorods on flexible Ti metal foil

**DOI:** 10.1039/c9ra09707d

**Published:** 2020-01-10

**Authors:** Ch. Ramesh, P. Tyagi, J. Kaswan, B. S. Yadav, A. K. Shukla, M. Senthil Kumar, S. S. Kushvaha

**Affiliations:** CSIR-National Physical Laboratory Dr K. S. Krishnan Road New Delhi India 110012 kushvahas@nplindia.org; Academy of Scientific and Innovative Research (AcSIR) Ghaziabad India 201002; Solid State Physics Laboratory Lucknow Road, Timarpur Delhi India 110054

## Abstract

The effect of flexible Ti metal foil surface modification and laser repetition rate in laser molecular beam epitaxy growth process on the evolution of GaN nanorods and their structural, electronic and optical properties has been investigated. The GaN nanostructures were grown on bare- and pre-nitridated Ti foil substrates at 700 °C for different laser repetition rates (10–30 Hz). It is found that the low repetition rate (10 Hz) promotes sparse growth of three-dimensional inverted-cone like GaN nanostructures on pre-nitridated Ti surface whereas the entire Ti foil substrate is nearly covered with film-like GaN consisting of large-sized grains for 30 Hz growth. In case of the GaN growth at 20 Hz, uniformly-aligned, dense (∼8 × 10^9^ cm^−2^) GaN nanorods are successfully grown on pre-nitridated Ti foil whereas sparse vertical GaN nanorods have been obtained on bare Ti foil under similar growth conditions for both 20 and 30 Hz. X-ray photoemission spectroscopy (XPS) has been utilized to elucidate the electronic structure of GaN nanorods grown under various experimental conditions on Ti foil. It confirms Ga–N bonding in the grown structures, and the calculated chemical composition turns out to be Ga rich for the GaN nanorods grown on pre-nitridated Ti foil. For bare Ti substrates, a preferred reaction between Ti and N is noticed as compared to Ga and N leading to sparse growth of GaN nanorods. Hence, the nitridation of Ti foil is a prerequisite to achieve the growth of dense and aligned GaN nanorod arrays. The X-ray diffraction, high resolution transmission electron microscopy and Raman studies revealed the *c*-axis growth of wurtzite GaN nanorods on Ti metal foil with good crystallinity and structural quality. The photoluminescence spectroscopy showed that the dense GaN nanorod possesses a near band edge emission at 3.42 eV with a full width at half maximum of 98 meV at room temperature. The density-controlled growth of GaN nanorods on a flexible substrate with high structural and optical quality holds promise for potential applications in futuristic flexible GaN based optoelectronics and sensor devices.

## Abstract 2

The effect of flexible Ti metal foil surface modification and laser repetition rate in laser molecular beam epitaxy growth process on the evolution of GaN nanorods and their structural, electronic and optical properties has been investigated.

## Graphical abstract

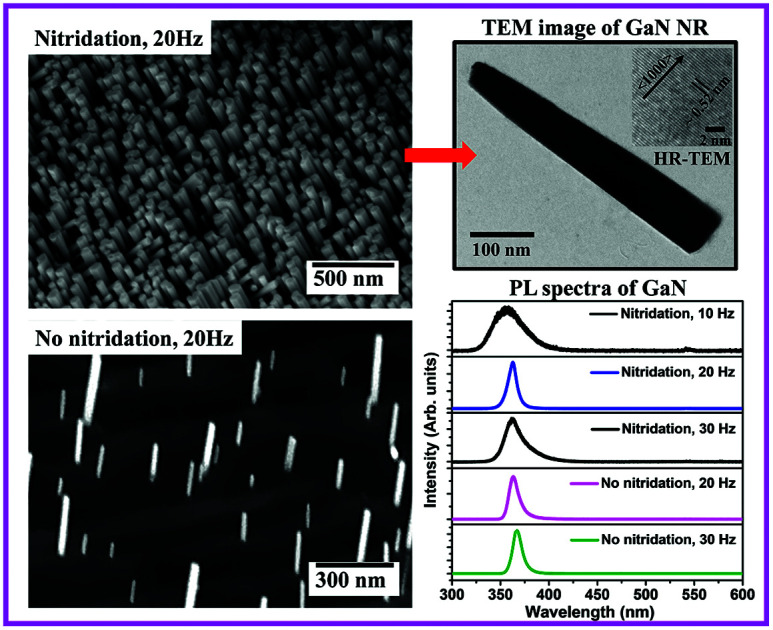

## 1. Introduction

Wide and direct band gap GaN and its alloys with Al and In metals have long been considered as excellent semiconductors for applications over a broader wavelength range from ultra-violet to deep-blue light emitting diodes (LEDs), photo-detectors, high electron mobility transistors and laser diodes.^1–5^ Due to the unavailability of large-sized GaN single crystals or wafers, the conventional sapphire and silicon based substrates are utilized for the hetero-epitaxial growth for the fabrication of GaN based devices.^4,6^ However, there is a large thermal and lattice mismatch between these substrates and GaN which results in a high dislocation density and residual stress in the grown layers.^7,8^ The growth of one-dimensional (1-D) GaN nanowires and nanorods is one of the alternative approaches to reduce the defects/stress as reported by several research groups.^9–11^ Even though the dislocation lines present in nanowires cannot be eliminated they are mostly constrained at the interface of substrates. Thanks to the high aspect ratio of nanowires, stacking faults and threading dislocation (TD) density in the upper part of nanowires are reduced as TDs terminate at the sidewall of nanowires.^12–14^ The advantage of GaN nanowire/nanorod growth includes the improvement of light extraction efficiency, suppression of quantum confined Stark effect and increase of active LEDs device area.^13,15^

Several researchers have reported the growth of GaN nanowires on conventional substrates such as sapphire, silicon and metal coated sapphire using plasma assisted molecular beam epitaxy (PA-MBE).^16–19^ For the advancement of LED devices, there is a high demand to grow GaN nanowires on flexible substrates for futuristic applications in wearable intelligent devices, rollable displays and lighting devices.^12,13^ There are various flexible substrates such as polymer based indium tin oxide coated substrates, plastic films and organic materials but the growth of III-nitrides on these substrates is limited due to their low melting temperature. Recently, thin metal foils are being considered as alternate flexible substrates for the growth of GaN nanowires because of their excellent thermal and electrical conductivities along with a high optical reflectance, which open up unique possibilities of developing flexible GaN based devices.^20,21^ A few groups have recently reported the growth of GaN nanowires on Ti and Ta foils using PA-MBE at growth temperature of 730–800 °C.^12,13^ Here, Ti metal foil has an added advantage as it can serve as the direct bottom contact to GaN. The growth of GaN nanowires on flexible and reactive metal foils at relatively high growth temperature of 800 °C using conventional MBE process may lead to the formation of unwanted interfacial alloys or compounds. In view of this, laser assisted molecular beam epitaxy (LMBE) offers the relatively low temperature process for GaN growth due to the highly energetic GaN precursors produced by laser ablation of target materials.^21^ May *et al.* have obtained high density aligned growth of GaN nanowires on bare Ti foil in which, the individual grains of polycrystalline Ti foil influenced the tilt and density of the grown nanowires.^12^ On the other hand, Calabrese *et al.* has grown GaN nanowires on pre-nitridated Ti foil that are largely aligned normal the substrate surface.^13^ In the following report, they found that the presence of an amorphous oxide layer on pre-nitridated Ti foil promoted vertically well-aligned growth of GaN nanowires all across the substrate as it prevented the transfer of substrate information for any epitaxial growth.^20^ Thus, the role of Ti nitridation appears to be very critical in determining the alignment and density of GaN 1D structures but demands further knowledge to establish the process. Keeping in view of this, here, we studied the effect of surface nitridation of Ti foil over the formation of GaN 1D structure with the help of electronic structure as analyzed by X-ray photoelectron spectroscopy (XPS). In addition, we also explore the role of laser repetition rate on the size and density of GaN nanostructures by employing LMBE technique. It is found that the GaN growth using a moderate laser repetition rate of 20 Hz offers a dense and aligned GaN nanorod array on pre-nitridated Ti metal foils but sparse and vertically grown individual GaN nanorods on bare Ti foils. The XPS analysis indicates that the surface of Ti foil is more reactive to N radicals as compared to Ga, which leads to sparse growth of GaN nanorods on bare Ti foil. Whereas, the pre-nitridation of Ti surface induces an inclined growth of dense nanorods whose chemical composition turns out to be Ga rich.

## 2. Experimental

Self-assembled catalyst-free GaN nanostructures were grown on a 0.127 mm thick Ti metal foil (Alpha Aesar, 99.99% pure) using LMBE (base pressure: 2 × 10^−10^ torr) technique. The Ti metal foils were cleaned using standard organic solvents and transferred to a preparation cum load lock chamber after dried with nitrogen gas. The Ti foil was degassed for few hours at 200 °C in the load lock chamber followed by thermal cleaning at 850 °C for 30 min in the UHV growth chamber using infra-red radiation resistive heater. The high temperature (HT) nitridation of Ti foil surface was performed at 850 °C under r.f. nitrogen plasma having a power of 400 W and semiconductor grade high pure nitrogen gas flow of 1.1 sccm for 20 min. A high purity hydride vapor phase epitaxy (HVPE) grown polycrystalline solid GaN target (99.9999%) was ablated using a KrF excimer laser (wavelength: 248 nm) in the presence of additional supply of r.f. nitrogen plasma (250 W r.f. power with 0.4 sccm nitrogen flow). GaN was grown on pre-nitridated Ti metal foils at 700 °C for 2 h by varying the laser repetition rates from 10 to 30 Hz, keeping other parameters constant. To see the effect of pre-nitridation, the GaN was also grown on bare Ti metal foil at the laser repetition rate of 20–30 Hz under similar growth parameters.

The surface morphology of the LMBE grown GaN nanostructures was characterized using field emission scanning electron microscopy (FE-SEM) in 45° tilt-view at an operating voltage of 5 kV. A high resolution X-ray diffraction (HR-XRD) system was employed to characterize the crystalline nature of LMBE grown GaN on Ti foils using CuKα_1_ source. The high-resolution transmission electron microscopy (HR-TEM) study was performed at an operating voltage of 300 kV on the GaN nanorods dispersed on Cu grid. The room temperature micro-Raman spectra were measured in back-scattering geometry using a laser as an excitation source (*λ* = 514.5 nm). The optical properties were characterized using PL spectroscopy using a semiconductor laser source with an excitation wavelength of 266 nm at room temperature. Non-monochromatic dual anode (Al K_α_ and Mg K_α_) and monochromatic Al K_α_ X-ray sources and EA125 electron analyzer from Omicron GmbH have been used to perform XPS measurements. C 1s (284.8 eV) core-level binding energy (BE) is used for charging correction and conducting Ag paint has been used to make electrical contact between sample surface and grounded sample plate. BE calibration has been done by using Fermi edge of an Ar^+^ sputtered clean polycrystalline Ag sample. Overall experimental energy resolution with 20 eV analyzer pass energy for monochromatic Al Kα (1486.7 eV) and non-monochromatic Mg Kα (1253.6 eV) is 0.45 and 0.9 eV, respectively. Uncertainty in determining the BE position and full width at half maximum (FWHM) is estimated to be ±0.1 eV. Mixture of Gaussian and Lorentzian (largely Gaussian) peaks has been used to fit the core level spectra and Shirley method has been used to remove secondary electron background.^22^

## 3. Results and discussion

**Fig. 1:**
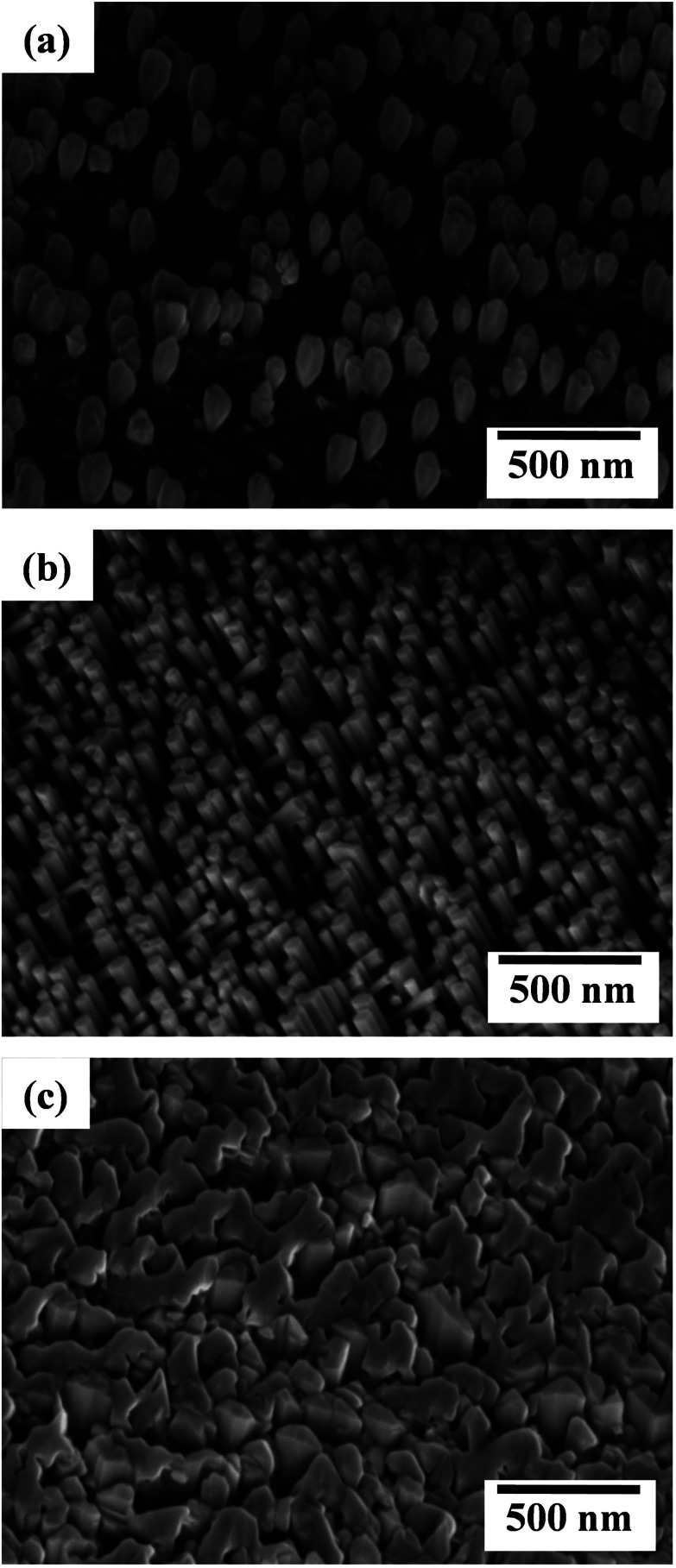
FESEM images (45° tilt-view) of GaN grown on HT-nitridated Ti metal foils at the growth temperature of 700 °C under various laser repetition rates: (a) 10 Hz, (b) 20 Hz and (c) 30 Hz.

Fig. 1(a) shows the 45° tilt-view FE-SEM image of LMBE grown GaN at 700 °C on HT pre-nitridated Ti foil with the laser repetition rate of 10 Hz. The three-dimensional (3D) conical-shaped irregular GaN islands were obtained on the Ti surface. The statistical analysis revealed that the height of the conical islands is in the range of 220–330 nm. The growth of GaN islands occurs due to N-rich growth condition (N/Ga ratio > 1) which mostly favors 3D GaN growth.^23^ With further increase of laser repetition rate to 20 Hz, the Ti foil is mostly covered with uniformly aligned, hexagonally-faceted GaN nanorods as shown in Fig. 1(b). The nanorods are largely un-coalesced and the size analyses indicate that the length and diameter of the nanorods are in the range of 400–500 and 50–60 nm, respectively. The density of the GaN nanorods was estimated by using several FE-SEM images and it is nearly ∼8 × 10^9^ cm^−2^ which is higher than GaN nanorods grown on low temperature (700 °C) nitridated Ti foil.^21^ For the growth at 30 Hz, larger GaN islands were obtained on Ti foil as shown in Fig. 1(c). The irregularly shaped islands have a lateral width in the range of 400–600 nm. These findings disclose the effect of laser repetition rate on the surface morphology of GaN nanostructures on nitridated Ti metal foils and a moderate repetition rate of 20 Hz is found to promote the growth of oriented GaN nanorods with a high areal density. Comparing with our previous studies, it is noted that the density of GaN nanorods on pre-nitridated Ti foils increased about one order with the increase of pre-nitridation temperature from 700 to 850 °C.^21,24^

**Fig. 2:**
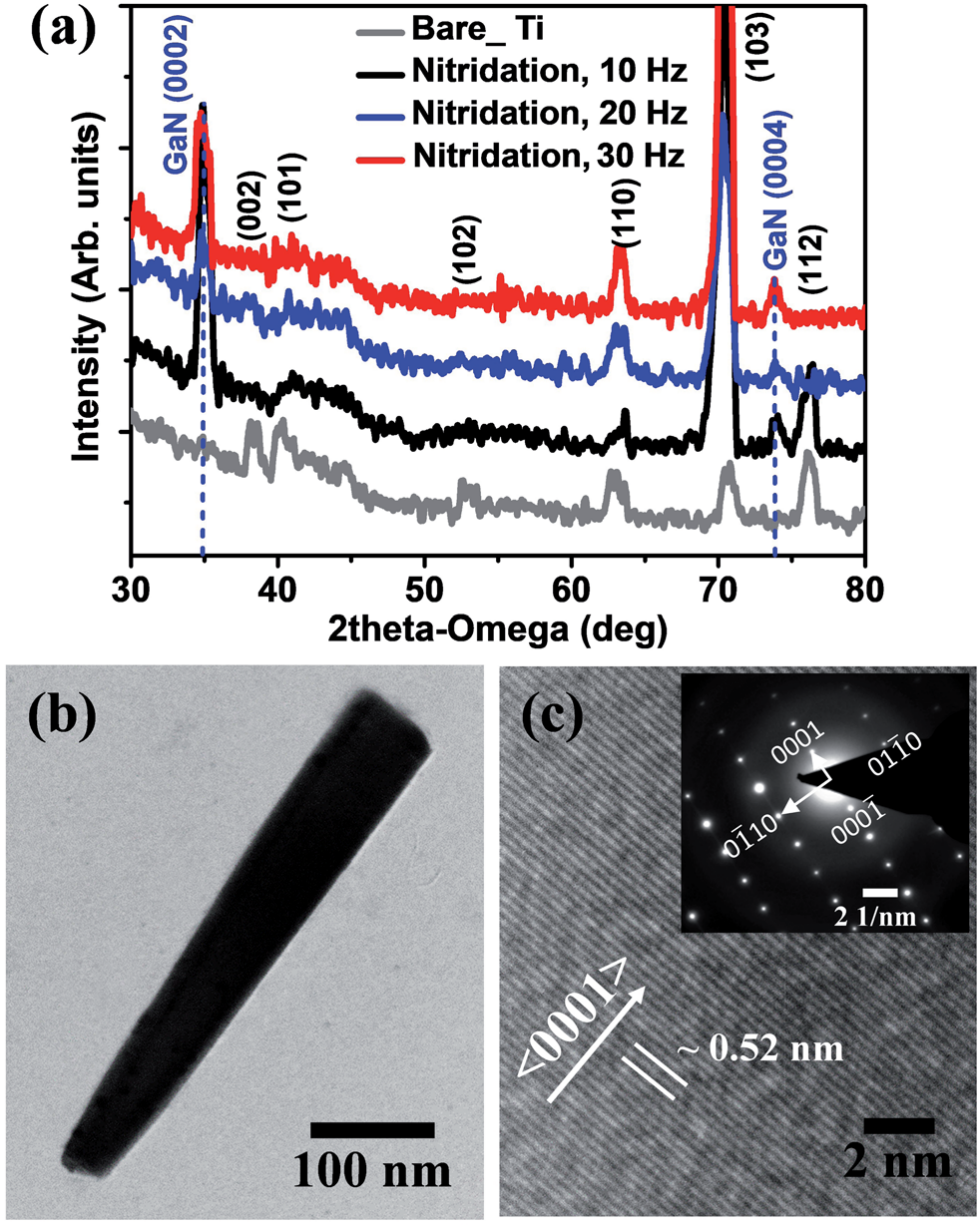
(a) 2*θ*–*ω* XRD pattern of bare Ti foil and LMBE grown GaN NRs on HT-nitridated Ti metal foil grown at different laser repetition rates. (b) Bright-field TEM and (c) HR-TEM micrographs of GaN nanorod grown on nitridated Ti metal foil at 20 Hz. Inset in (c) shows the SAED pattern.

The crystalline structure of the LMBE grown GaN nanostructures on nitridated Ti foil at different laser repetition rates and Ti foil substrate were characterized using 2*θ* XRD scan and it is presented in Fig. 2(a). The XRD scan showed the cubic structure of Ti having various planes such as (002), (101), (102), (110), (103) and (112) as denoted in the Fig. 2(a).^21^ The XRD peak around 45° is assigned to TiO_2_ (112) plane. In addition, the wurtzite GaN structure of (0002) and (0004) planes were also seen along with Ti foil peaks. After GaN growth, the Ti (103) peak intensity noticeably increased implying the recrystallization of the respective grains during the process. The XRD patterns indicate that the LMBE GaN nanostructures on Ti metal foil are grown along *c*-axis direction. In addition, the TEM study was also performed on the GaN nanorods grown at 20 Hz. The GaN nanorods were dispersed on the Cu grid and bright field TEM is presented in Fig. 2(b). The inversely-tapered GaN nanorod with a length of 450 nm is clearly observed, which complimented the FE-SEM result. The HR-TEM image taken on top of GaN nanorods is presented in Fig. 2(c) and it shows the clear lattice arrangements without any stacking faults. The measured closest inter-planar distance (lattice spacing) is ∼0.26 nm which corresponds to the GaN (0001) crystal structure. The respective selective area electron diffraction (SAED) pattern is included as the inset of Fig. 2(c). These observations revealed that the *c*-axis oriented GaN NR has been grown on Ti metal foils, similar to the previous reports.^12,21^

**Fig. 3:**
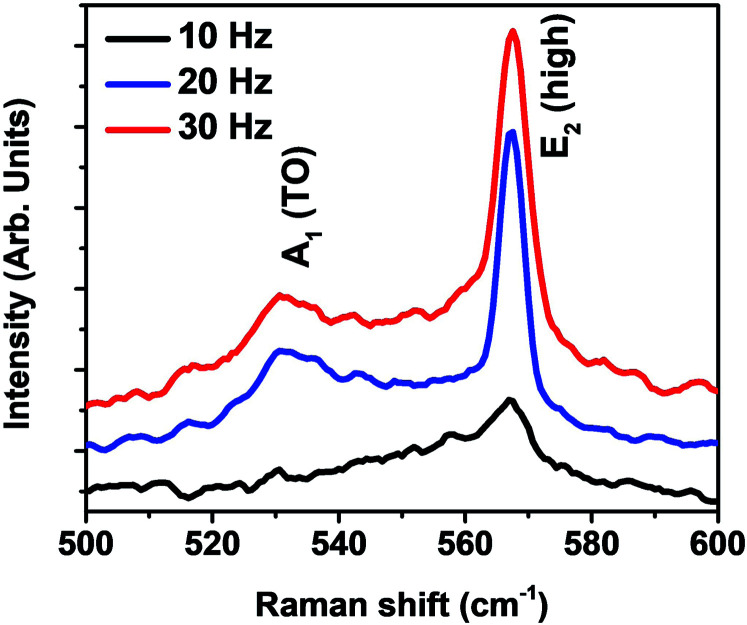
Raman spectra of GaN nanostructures grown on nitridated Ti foil at laser repetition rates of 10, 20 and 30 Hz.

Fig. 3 represents the room temperature Raman spectra of the GaN nanostructures grown on HT-nitridated Ti foil. A dominant Raman E_2_ (high) peak has been observed in the Raman spectra in comparison to other active Raman modes which is the characteristics of wurtzite structure of GaN.^25–28^ In addition, A_1_ (TO) peak has been observed at around 532.09 and 531.19 cm^−1^ for the GaN grown at 20 and 30 Hz, respectively. The E_2_ (high) peak position and FWHM value describe the structural quality of GaN nanostructures and are estimated by Lorentzian fitting of the Raman spectrum.^28^ The peak position (FWHM) of the E_2_ (high) for LMBE grown GaN on Ti foil is 567.01 (7.65), 567.34 (3.66) and 567.49 (5.01) cm^−1^ for samples grown at laser repetition rate of 10, 20 and 30 Hz, respectively. Compared with the E_2_ (high) peak position of stress free thick GaN film (567.6 cm^−1^), the obtained values shows only a slight peak shift that indicates the growth of nearly stress-free GaN on Ti foil.^26,27^ The low FWHM value of E_2_ (high) mode for GaN nanorods manifests the good structural quality of the wurtzite GaN nanorods at 20 Hz.

**Fig. 4:**
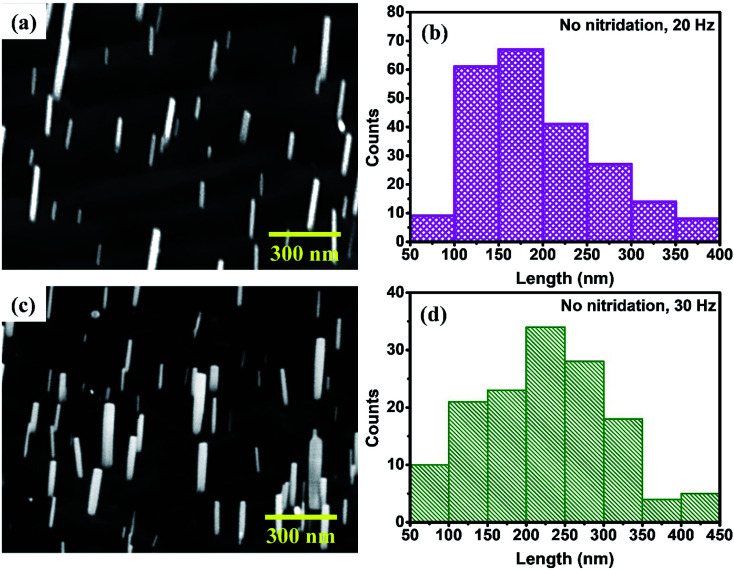
45° tilt-view FESEM images of GaN nanorods grown on bare Ti foil at (a) 20 Hz and (c) 30 Hz; (b) and (d) are the respective histogram of GaN nanorod length.

In order to examine the effect of Ti pre-nitridation, few samples were also grown on thermally cleaned Ti foil without nitridation. Fig. 4(a) and (b) represent the 45° tilt-view FESEM image of GaN nanorods grown on bare Ti metal foil at 700 °C with the laser repetition rate of 20 Hz and the histogram of nanorod length, respectively. Sparse nanorods of density in the range ∼5.5 × 10^8^ cm^−2^ are grown nearly perpendicular to the Ti foil substrate, having the diameter of 20–60 nm. The length of the nanorods varied mostly in the range of 100–300 nm. Sparse GaN nanorods were also grown on bare Ti foil at laser repetition rate of 30 Hz as shown in Fig. 4(c), whereas, the coalesced large 3D island-like GaN film was seen on nitridated Ti foil [Fig. 1(c)]. From the statistical analyses and histogram presented in Fig. 4(d), the density, diameter and length of the GaN nanorods are ∼7 × 10^8^ cm^−2^, 40–80 nm and 100–350 nm, respectively. The histogram presented in Fig. 4(b) and (d) are plotted after statistical analysis of several FESEM images and the nanorods obtained on bare Ti foil for 20 and 30 Hz with length above 450 nm is very few. These observations revealed that there is no significant increase in size and density for nanorods grown on bare Ti foil at 30 Hz. The energy dispersive X-ray (EDX) analysis was carried out for GaN nanorods grown on nitridated and bare Ti foils and it was found that the atomic percentage of Ga and N turn out to be 46.4 ± 0.5 and 53.6 ± 0.5 for dense GaN nanorods whereas, Ga and N atomic percentages were found to be 30.6 ± 0.5 and 69.4 ± 0.5 for sparse GaN nanorods, respectively.

**Fig. 5:**
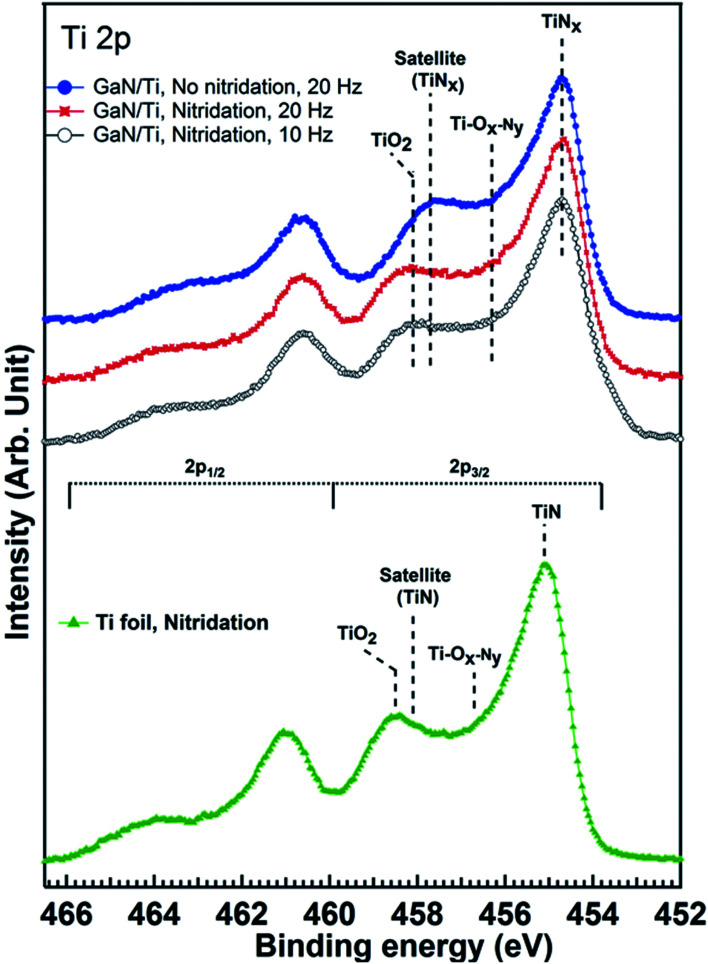
Ti 2p core level spectra from nitridated Ti metal foils with and without GaN growth. XPS core levels shown here have been acquired using monochromatic Al K_α_ X-ray source. Each set of spectra has been normalized by the peak height of the highest peak and staggered along the *y* axis for clarity of presentation. Dashed lines indicate the peak positions of various species.

We have studied the electronic properties of LMBE grown sparse and dense GaN nanorods grown on Ti foil at different growth parameters. Lower panel of Fig. 5 exhibits Ti 2p_3/2_ and 2p_1/2_ core levels from nitridated Ti metal foil with spin orbit splitting of 5.9 eV. The BE main peak position (455.1 eV) along with corresponding shake-up satellite is characteristic of stoichiometric TiN which indicates towards efficient nitridation of Ti foil.^29–31^ Since sample has been transferred to XPS chamber after atmospheric exposure as it has been grown in a different chamber, we can also observe features related to oxide (TiO_2_: 458.5 eV) and oxy-nitride (Ti–O_*x*_–N_*y*_: 456.7 eV). Upper panel of Fig. 5 shows the set of Ti 2p core level spectra from Ti metal foils with GaN growth on bare and nitridated Ti foil and at different repetition rates. We can clearly see that all the core levels for GaN/Ti metal foil have similar BE position and these are shifted by 0.4 eV towards lower BE side compared to bare nitridated Ti foil. Such shift can be attributed to the formation of non-stoichiometric TiN_*x*_,^20^ and it evidences that Ti–N co-ordination of nitridated Ti foils get affected as GaN growth takes place. Similar shift is seen for oxide and oxy-nitride peaks as well. It is interesting to note that even in the case of GaN growth on Ti foil without prior nitridation (topmost spectrum of the upper panel), similar formation of TiN_*x*_ is observed as of GaN grown on nitridated foils. It suggests that formation of Ti–N precedes to GaN growth even if no prior nitridation has been performed. However, in case of no prior nitridation, a large portion of nitrogen which otherwise would have promoted Ga–N formation, will form Ti–N bond. In such scenario, Ga–N growth might be diminished, resulting in lesser nucleation sites, compared to growth on pre-nitridated Ti foils. It may be one of the causes for sparse growth of nanorods in this case, as shown above from our FESEM results. We also note that the degree of oxidation is different (higher) for sample grown without nitridation as larger part of this sample is exposed to ambient due to sparse growth of GaN nanorods.

**Fig. 6:**
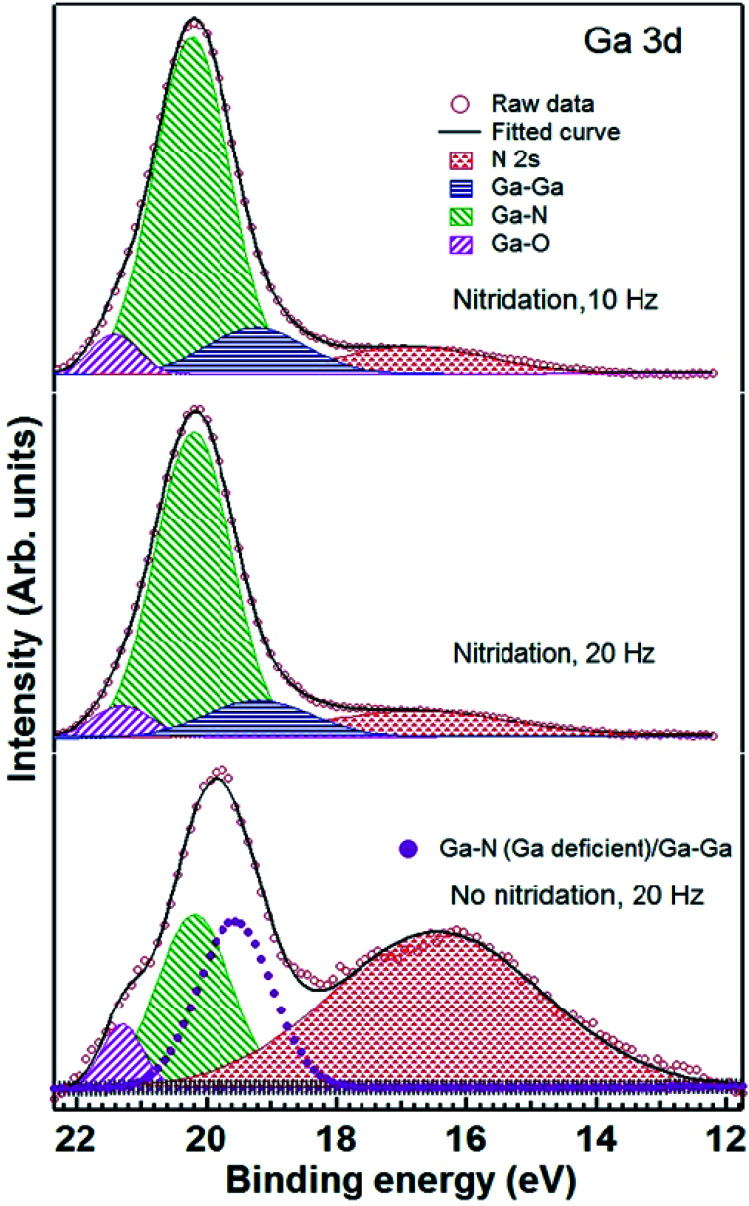
Ga 3d core level spectra of GaN grown on Ti foils with (10 and 20 Hz) and without (20 Hz) nitridation. Experimental data (open circles), fitted spectra (solid line), deconvoluted components used to fit Ga–N (green patterned), Ga–Ga (blue shaded), Ga–O (pink patterned) and N 2s (red patterned) are also shown. For GaN grown on Ti foil without nitridation, an extra component belonging to Ga deficient Ga–N/Ga–Ga is also shown (magenta filled circles). Spectra have been staggered vertically for the clarity of presentation.

Fig. 6 shows Ga 3d core level spectra for GaN grown on Ti foils with and without nitridation. Spectra for samples grown on nitridated Ti foils can be satisfactorily fitted using 3 components (Ga–O, Ga–N and Ga–Ga).^36^ Details of the fitting parameters are given in Table 1. N 2s related component is also shown here since it overlaps with the tail of Ga 3d core level. For both the samples, GaN grown on nitridated Ti foil using 10 and 20 Hz repetition rate, Ga 3d core level is almost similar with Ga–N component appearing at 20.2 ± 0.1 eV. However, for sample grown on Ti foil without nitridation, the shape of Ga 3d core level appears to be different and an additional component, centered at 19.6 eV, is required for the fitting. Since its energy position is different than the usual position (∼19.2 eV) expected for Ga–Ga component for Ga rich stoichiometry, it may be ascribed to Ga_*x*_N_*y*_ bonding in Ga deficient region along with overlapping contribution from unreacted Ga (Ga–Ga). This feature may also be linked to Ti–Ga intermixed species. We found higher Ga–O component for this sample, consequence of enhanced oxidation of the sample upon atmospheric exposure due to sparse nature of nanorod growth. Interestingly, we also observe large signal of N 2s related feature compared to samples grown on nitridated foils. It can once again be correlated to the probing of enhanced area of the substrate (TiN_*x*_) as it is sparsely covered by GaN nanorods.

**Table 1:** Summary of fitting parameters for Ga 3d (Fig. 6) and N 1s core level spectra (Fig. 7). Relative percentage of fitting components is calculated from the ratio of the area under individual peak and total peak area of all components. Uncertainty in determining the binding energy (BE) position and FWHM is estimated to be ±0.1 eV. Uncertainty in determining relative percentage is estimated to be ±5% of the base value

Sample	Nitridation, 10 Hz			Nitridation, 20 Hz			No Nitridation, 20 Hz		
Core level	Peak (BE position, eV)	FWHM (eV)	Relative%	Peak (BE position, eV)	FWHM (eV)	Relative%	Peak (BE position, eV)	FWHM (eV)	Relative%
Ga 3d	Ga–O (21.4)	0.9	5.0	Ga–O (21.3)	1.0	6.0	Ga–O (21.3)	0.8	9.0
	Ga–N (20.2)	1.4	81.0	Ga–N (20.2)	1.4	80.0	Ga–N (20.2)	1.3	47.0
	Ga–Ga (19.2)	1.8	14.0	Ga–Ga (19.2)	1.9	14.0	Ga–N (Ga deficient)/Ga–Ga (19.6)	1.4	44.0
N 1s	NH/NH_2_ (398.6)	1.9	10.0	NH/NH_2_ (398.7)	1.7	8.0	NH/NH_2_ (398.6)	2.1	12.0
	N–Ga (397.5)	1.3	46.0	N–Ga (397.5)	1.3	48.0	N–Ga (397.5)	1.3	18.0
	N–Ti (396.5)	1.2	32.0	N–Ti (396.5)	1.2	30.0	N–Ga (Ga deficient)/N–N (397.0)	0.9	22.0
	N–Ti–O (395.7)	1.35	12.0	N–Ti–O (395.7)	1.35	14.0	N–Ti (396.5)	1.25	39.0
							N–Ti–O (395.8)	1.4	9.0

**Fig. 7:**
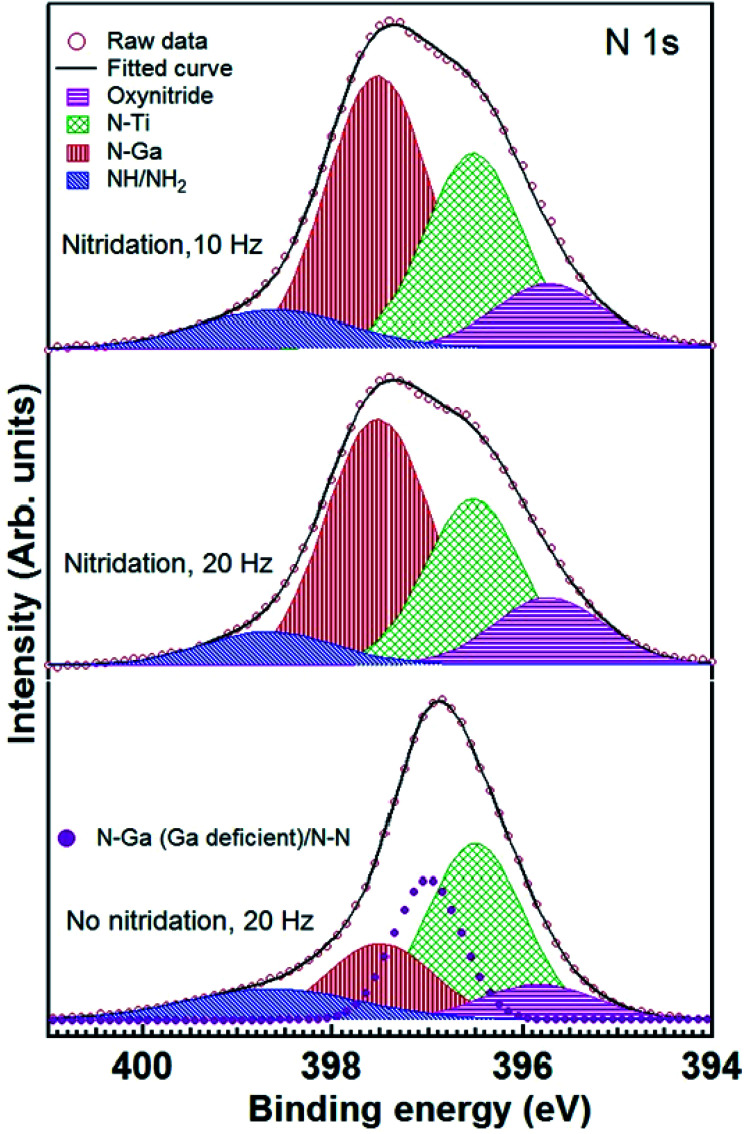
N 1s core level spectra of GaN grown on Ti foils with (10 and 20 Hz) and without (20 Hz) nitridation. Experimental data (open circles), fitted spectra (thick solid line), deconvoluted components used to fit N–Ga (red patterned), N–H + N–H_2_ (blue patterned), oxynitride (pink patterned) and N–Ti (green patterned) are also shown. For GaN grown on Ti foil without nitridation, an extra component belonging to Ga deficient N–Ga/N–N is also shown (magenta filled circles). Spectra have been staggered along the *y* axis for clarity of presentation.

Fig. 7 exhibits N 1s core level spectra for LMBE grown GaN on Ti foils with and without nitridation. N 1s core levels for GaN grown on nitridated Ti foils look similar and these can be fitted with 4 components corresponding to N–H/N–H_2_, N–Ga, N–Ti and TiO_*x*_N_*y*_ (oxynitride) species.^30–32^ Details of the fitting parameters are given in Table 1. However, for the GaN grown on Ti foil without prior nitridation, N 1s core level shape is quite different and an extra feature centered at 397.0 eV BE is required to fit N 1s core level satisfactorily. It may be ascribed to N–Ga bond in the Ga deficient region and/or homopolar N–N bonds. We have calculated Ga/N ration for all the samples after taking into account of photoemission cross section, inelastic mean free path and analyzer transmission function. Calculated Ga/N ration for GaN grown on pre-nitridated Ti foils using 10 and 20 Hz repetition rate turns out to be 1.3 ± 0.1 and 1.2 ± 0.1, respectively. However, interestingly, the Ga/N ratio for GaN grown on Ti foil without prior nitridation turns out to be 0.4 ± 0.05, indicating significant Ga deficiency. It is also evident form Table 1 that Ga–N/N–Ga relative percentage sharply decreases for GaN grown without nitridation. On the other hand, it is clear from Fig. 5 and Table 1 that the extent of Ti–N_*x*_ formation is found to be similar for the GaN grown with and without nitridation. It suggests that Ga/N ratio strongly depends on prior nitridation of the Ti foil and consequent Ti–N formation. Prior nitridation of Ti foil effectively makes available a Ti–N_*x*_ template and GaN growth proceeds normally. However, in the case of no prior nitridation, there is a competition between Ti–N_*x*_ and Ga–N formation and our XPS data clearly shows that Ti–N_*x*_ formation precedes Ga–N formation. In this case, since GaN plume and nitrogen radicals from plasma source arrive simultaneously at the Ti foil, nucleation sites for GaN growth become only available after formation of Ti–N_*x*_. Since formation of Ti–N_*x*_ is favored over Ga–N in the same growth time scale, a significant part of GaN plume arrived at the substrate does not nucleate which results in sparse GaN growth. Even though the Ga/N ratio and prior condition in terms of Ti–N_*x*_ growth template is similar for GaN growth at 10 and 20 Hz repetition rate with pre-nitridation, it is interesting to note that the growth morphology is quite different for both cases and it is related with the arrival rate of the GaN plume.

The growth of various GaN nanostructures on Ti metal foil with respect to laser repetition rate at the fixed growth temperature of 700 °C can be understood in terms of variation in surface diffusion of ad-atoms at different growth rates. It is well-known that, in MBE process, a 3D GaN growth occurs under N-rich flux condition whereas Ga-rich growth condition promotes the 2D thin film growth. Since the stoichiometric GaN target was ablated in the presence of additional nitrogen-plasma, it is likely to be N-rich growth condition.^21,23^ At the low flux rate, initially GaN adatom or cluster nucleates randomly at grains and/or grain boundaries on the Ti metal foil and further changed to spherical-shaped 3D islands. As growth proceeds, the rate of new nucleation becomes less and the impinging flux prefers to grow on the existing GaN islands or clusters. Consequently, the conical-shaped 3D islands are obtained on the Ti metal foil at the low flux rate due to the different surface diffusion rate of Ga-adatom along polar and non-polar GaN. In case of moderate flux, the initial nucleation of GaN adatom occurs on most of Ti metal foil since the growth rate is relatively higher. Later on, as the growth proceeds under moderate growth rate, the initially nucleated clusters or islands undergo shape transformation and propagate along vertical direction with {0001} facet on the top because *c*-axis planes have a low surface energy in wurtzite structure.^21^ The growth of self-induced GaN nanorods is highly influenced by growth rate during shape transformation and it is essential to overcome energy barrier which is sensitive to the sidewall surface energy.^33^ In case of high growth rate (30 Hz), the density of initial nucleation is relatively high and later on, the growth occurs in both lateral and vertical directions. Subsequently, the large, coalesced GaN island growth occurs on the nitridated Ti metal foil [Fig. 1(e and f)].

The dense and sparse GaN nanorods obtained, respectively, on nitridated and bare Ti metal foils are related with the reaction of N-radicals on Ti surface, which forms uppermost TiN_*x*_ layer rather stable GaN clusters on bare Ti foil. Calabrese *et al.* also reported that at lower growth temperature of 670 °C, the formation of GaN nanowires started 92 min after opening of Ga and N shutter simultaneously on bare Ti/Al_2_O_3_ surface and a formation of TiN and Ga-based hillocks at the interface was observed.^19^ At relatively high temperature of 690 °C, the formation of TiN is more prominent and the reduced lifetime of Ga adatom at 690 °C prevents the formation of any Ga-related hillocks on the Ti surface.^19^ In case of Ti metal foil, it has been reported that the impinging elements on bare Ti foil chemically react with Ti whereas the nitridated Ti foil consists of an amorphous oxide surface layer suppresses the influence of polycrystalline Ti substrate on GaN nanowire formation and facilitates the growth of uniformly oriented dense GaN nanowires.^20^ The XPS studies also support the formation of TiN with the presence of un-reacted Ga–Ga or partially intermixed Ga–Ti on top of Ti metal foils under the GaN growth on bare Ti at 700 °C. These observations revealed that the pre-nitridation of Ti metal foil is one of the decisive parameters for the growth of dense GaN nanorods.^19,20^ It should be noted that the GaN growth on bare Ti foil yielded uniform, vertical alignment of GaN nanorod over the substrate, however, the inclined growth of GaN nanorod is obtained on pre-nitridated Ti foil. It implies that there is a possible epitaxial relation between the nitridated surface layer and GaN. The nature of interfacial layer formed by surface nitridation is very critical to decide the alignment of growing nanorods. In the current case, the nitridated surface layer remains crystalline and transfers the substrate information for the formation of epitaxial GaN nanorod.

**Fig. 8:**
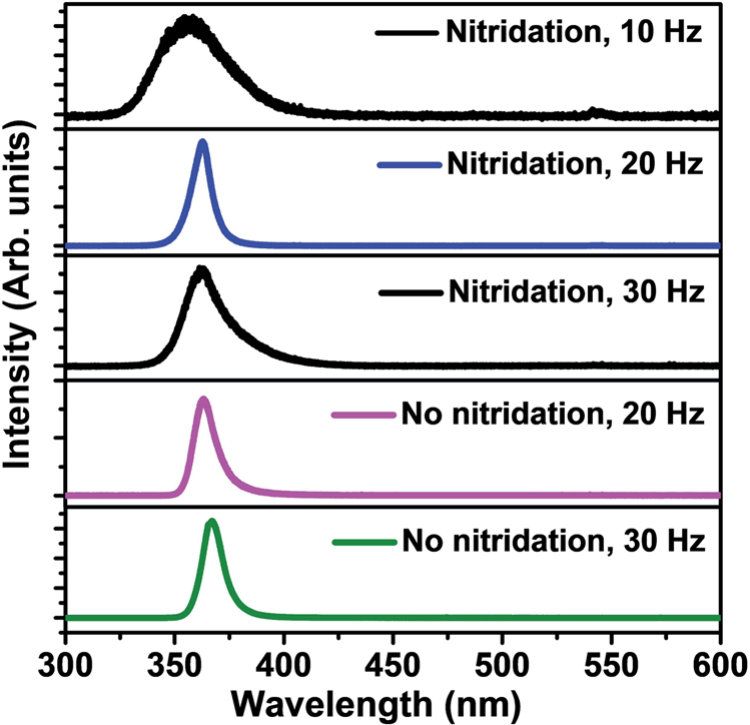
PL spectra of GaN nanostructures grown on bare and nitridated Ti foil at different laser repetition rates.

The PL spectra of LMBE grown GaN on bare and pre-nitridated Ti foils with different laser repetition rates are presented in Fig. 8. Wurtzite GaN generally exhibits the near band edge (NBE) emission peak at ∼3.4 eV at room temperature and the defect related PL peaks in the range of 1.4–2.8 eV. Among defect related emissions, the yellow luminescence (YL) band at ∼2.1–2.4 eV is more common in GaN due to the complex crystalline defects.^34^ For GaN nanorods grown on bare and pre-nitridated Ti foil NBE emission peak is obtained in ∼3.38–3.46 eV without any emission line associated to broad YL defect band. After Lorentzian fitting of PL spectra, the FWHM values for GaN samples grown at 10, 20 and 30 Hz on pre-nitridated Ti foil are obtained at 360, 98 and 207 meV, respectively. The broader NBE FWHM of the GaN sample grown at 10 Hz can be related with the defect-related impurities present in 3D GaN. The optical property of sparse GaN nanorods grown on bare Ti foil using 20 and 30 Hz reveals the intense and sharp NBE peak position with the FWHM value of 110 and 96 meV, respectively. The NBE FWHM value of ∼96–98 meV for GaN nanorods grown on Ti foil is comparable to the PL emission of GaN nanowires grown on gold-coated Si substrate by MOCVD and sapphire (0001) substrate by simple catalyst CVD growth.^35,36^ The narrow NBE PL spectrum without any deep level defect band implies the excellent optical quality of the LMBE grown GaN nanorods on bare and nitridated Ti foil. The control of shape, size and density of GaN nanostructures in self-assembly process is one of the desirable requirements for futuristic nanoscale device fabrications and the size and density of LMBE grown GaN nanorods on Ti foil were tuned by the surface modification of foil and laser repetition rate.

## 4. Conclusions

The role of surface nitridation of Ti foil and laser repetition rate (10–30 Hz) on the formation, structural, optical and electronic properties of GaN nanostructures grown on Ti metal foil in LMBE growth process has been systematically studied. Cone-shaped sparse 3D islands were obtained on Ti foil at growth temperature of 700 °C with low repetition rate of 10 Hz whereas a dense (∼8 × 10^9^ cm^−2^) uniformly oriented GaN nanorod array of length and diameter in range of 400–500 and 50–60 nm were obtained, respectively, at the repetition rate of 20 Hz. The sparse, vertically-oriented GaN nanorods were obtained on the bare Ti metal foil at growth temperature of 700 °C with repetition rates of 20 and 30 Hz as N radicals are more reactive with Ti as compared to Ga, which leads to the sparse growth of GaN nanorods. The HRTEM and Raman studies revealed *c*-axis oriented, single crystalline growth of wurtzite GaN nanorods on Ti metal foil using LMBE technique. The XPS measurements confirm Ga–N bonding and the calculated chemical composition turns out to be slightly Ga rich for dense GaN nanorods. It is observed that the surface nitridation of Ti foil is essential to obtain a high density, aligned GaN nanorod array. The optical properties of GaN nanorods were measured using PL spectroscopy at room temperature and showed a strong NBE peak emission with the FWHM value of 96–110 meV. The controlled growth of dense and sparse GaN nanorods on surface modified flexible Ti metal foils can pave a way for the advancement in developing futuristic flexible and wearable III-nitrides based devices.

## Conflicts of interest

There are no conflicts to declare.

## Supplementary Material
